# Area-level and family-level socioeconomic position and body composition trajectories: longitudinal analysis of the UK Millennium Cohort Study

**DOI:** 10.1016/S2468-2667(21)00134-1

**Published:** 2021-07-29

**Authors:** Charis Bridger Staatz, Yvonne Kelly, Rebecca E Lacey, Rebecca Hardy

**Affiliations:** a Social Research Institute, Institute of Education, University College London, London, UK; b Department of Epidemiology and Public Health, University College London, London, UK

## Abstract

**Background:**

Inequalities in the trajectories of body composition in childhood and adolescence have been infrequently studied. Despite the importance of environmental factors in obesity development, little research has looked at area-level socioeconomic position, independent of family socioeconomic position. We aimed to assess how inequalities in body composition develop with age.

**Methods:**

The Millennium Cohort Study is a longitudinal study of 19 243 families who had a child born between 2000 and 2002 in the UK. Multilevel growth curve models were applied to examine change in fat mass index (FMI), fat free mass index (FFMI; using the Benn index), and fat mass to fat free mass ratio (FM:FFM), measured using Bioelectrical Impedance Analysis, from ages 7 years to 17 years by the Index of Multiple Deprivation (IMD) and household income at baseline.

**Findings:**

Inequalities in FMI and FM:FFM ratio are evident at age 7 years and widen with age. At age 17 years, adolescents in the most disadvantaged IMD group had FMI 0·57 kg/m^B^ (B=Benn parameter; 95% CI 0·43 to 0·70) higher and FM:FFM ratio 0·037 (95% CI 0·026 to 0·047) higher compared with the most advantaged group. Disadvantaged socioeconomic position is associated with higher FFMI but is reversed in adolescence after adjustment for FMI. Inequalities were greater in girls at age 7 years (mean FMI 0·22 kg/m^B^; 95% CI 0·13 to 0·32) compared with boys of the same age (0·05 kg/m^B^; –0·04 to 0·15, p=0·3), but widen fastest in boys, especially for FMI, in which there was over an 11 times increase in the inequality from age 7 years of 0·05kg/m^B^ (95% CI –0·04 to 0·15) to 0·62 kg/m^B^ at 17 years (0·42 to 0·82). Inequalities for the IMD were similar to income, and persisted at age 17 years independent of family socioeconomic position.

**Interpretation:**

Childhood and adolescence is an important period to address inequalities in body composition, as they emerge and widen. Policies should consider FFM as well as FM, and inequalities in the environment.

**Funding:**

Medical Research Council, Economic and Social Research Council.

## Introduction

Systematic reviews have shown consistent associations between disadvantaged socioeconomic position (SEP) and higher body-mass index (BMI) among children in high-income countries, including the UK.^1^, ^2^ Socioeconomic position is an umbrella term that captures social circumstances and can be measured by a number of indicators such as income, education, occupation, and area-level deprivation. Understanding the unequal burden of obesity according to SEP is important, as public health interventions aimed at reducing obesity that do not take into account inequalities could inadvertently increase them.^3^ Although there is clear evidence in relation to inequalities in BMI, less research has investigated inequalities in body composition, which better represents health risk because central fat mass (FM),^4^ and total fat free mass (FFM)^5^ are associated with cardiometabolic health.

Among adolescents (12–17 years), lean body mass is positively related to cardiovascular risk among girls, independent of FM but not of height.^6^ Additionally, FFM in adulthood is positively related to cardiovascular capacity, which in turn is inversely related to cardiovascular risk.^7^ Proportions of FFM in older age (60 years and older) are dependent on peak FFM and age-related decline, with peak FFM being reached in earlier adulthood (late thirties), unlike FM which increases into older age.^8^, ^9^ The amount of FFM has been shown to track from childhood to adulthood,^10^ but it is unclear whether socioeconomic inequalities in FFM also track across the life course. Our previous systematic review found evidence that disadvantaged SEP was associated with greater FM and lower FFM in high-income countries, but found few studies indexing measures to height^11^ and highlighted a paucity of studies using area-level measures of SEP.

Findings from systematic reviews showed that measures of area-level SEP were related to obesity measured by BMI.^1^, ^2^ Studies in the UK using area-level SEP in relation to body composition have reported mixed results.^12^, ^13^, ^14^ Although area-level SEP has often been used as a proxy for individual circumstances, it is likely to also capture broader aspects, specifically social and economic aspects, of the environment in which people live. Conceptual models have highlighted how neighbourhood deprivation relates to obesity through the built and social environment.^15^ The 2007 Foresight Report listed deprivation as a core measure of the environment, and one that needs greater research.^16^ Since social inequalities in adiposity emerged with the onset of a more obesogenic environment, there is a need to distinguish the influence of area and individual measures of SEP, to understand the role of neighbourhood environments beyond individual circumstances, and to ensure policy is effective in reducing obesity.^17^

**Research in context**

**Evidence before this study**

This research follows on from a systematic review that we previously conducted, investigating associations between socioeconomic position and body composition in children. In this study, we searched three databases (MEDLINE, Embase Classic+Embase, and SPORTDiscuss) from earliest entry until Jan 30, 2019, for literature published in English, investigating the association between socioeconomic position and body composition. Search terms covered body composition (eg, fat mass, muscle mass, etc), measurement techniques for body composition (eg, bioelectrical impedance) and socioeconomic position (eg, income, education, etc); full details are in the protocol. We identified 50 papers of childhood; 34 from high-income countries, and six unique studies from the UK. In high-income countries, disadvantage is shown to be associated with higher fat mass (FM) and lower fat free mass (FFM). Few studies reported on differences by sex in FFM in high-income countries. Most previous studies looked at raw or percentage measures of body composition, and only one study reported on a ratio measure. A range of socioeconomic position measures were considered, finding consistent results, but there was little evidence available for income and area-level socioeconomic position. We also considered evidence from studies looking at trajectories of body-mass index and body composition across childhood in high-income countries, with those identified finding increases in inequalities across childhood.

**Added value of this study**

This study adds value by looking at the trajectories of three measures of body composition (fat mass index, fat mass index [FFMI], and their ratio) over 10 years across childhood and adolescence in a nationally representative sample in the UK, enabling an investigation of how inequalities in body composition develop with age. Previous evidence of socioeconomic inequalities in FFMI and FM:FFM ratio in high-income countries has been sparse. We also used indexed measures of FM and FFM, calculating age and sex specific Benn parameters, which allowed FM and FFM to be interpreted independently of each other, and accounts for the correlation of mass with height. We show that the associations between socioeconomic position and FFMI differ to associations observed among studies using percentage or raw measures, indicating that inequalities in height might be an important contributing factor to inequalities in FFM. This study also distinguishes between area-level and family-level measures of socioeconomic circumstances and attempts to isolate the effect of area deprivation on body composition.

**Implications of all the available evidence**

There has been substantial attention directed towards tackling obesity in childhood, but greater policy consideration should be given to tackling inequalities in body composition in early life, including FFM, which peaks at an earlier age than does FM. Steps should be taken earlier to avert the need for more intensive public health interventions at older ages, especially for FFM in which such measures might be less effective at reducing inequalities, as they will rely on preventing inequalities in age-related decline in FFM instead of promoting peak FFM. Particular focus should be addressed to the socioeconomic environment, which this study shows has an independent effect on body composition in adolescence. Future research should continue to follow up existing cohort studies to observe if widening of inequalities in body composition continues across adulthood, and to track sex differences in the trajectories. We also suggest that greater consideration is needed in the selection of body composition measures, in the discussion of their strengths and weaknesses, and in separating the effects of area and family deprivation.

Women typically have lower FFM and higher FM than do men.^8^ Sex differences in inequalities in BMI have been well described in children,^1^, ^18^ but whether these differences in inequalities of body composition in children are observed, especially in relation to FFM, is unclear.^11^ Understanding how and when inequalities emerge in childhood, and whether there are sex differences, is important to fully understand the public health implications of inequalities in body composition in later life. This knowledge is particularly important for inequalities in FFM because the early peak means that inequalities evident in childhood and persisting through to adulthood might be more difficult to address in older age.

We use the Millennium Cohort Study, a nationally representative, ethnically diverse, birth cohort that has collected body composition at four timepoints (ages 7 years, 11 years, 14 years, and 17 years) to investigate inequalities according to family SEP, indicated by family income as a direct measure of access to material resources, and area-level SEP in FM, FFM, and their ratio. We investigate differences by sex and assess the extent to which area inequalities are independent of family SEP. We hypothesise that disadvantaged SEP is related to higher FM and lower FFM at age 7 years and that these inequalities widen with increasing age, that inequalities are greater in females compared with males, and that area-level inequalities are independent of individual SEP.

## Methods

### Dataset

The Millennium Cohort Study is a longitudinal study of 18 552 families (18 827 children) with children born between 2000 and 2002 in the UK and recruited at 9 months of age if eligible for the almost universal child benefits scheme.^19^ At age 3 years, recruitment of 692 new eligible families occurred bringing the total number of children to 19 517 (19 243 families). The sample was carefully constructed to have a large sample size and full representation of the UK population, including children from disadvantaged circumstances, minority ethnic groups, and the smaller countries of the UK (ie, Wales, Scotland, and Northern Ireland), which were oversampled, therefore allowing analysis with substantial statistical power within subgroups. Thus far, seven sweeps have occurred at 9 months, and years 3, 5, 7, 11, 14 and, most recently, at age 17 years in which 10 625 families took part. The analytical sample is limited to the first cohort member in each family, to ensure independence of observations and to prevent clustering by family. Ethics approval was obtained by the National Health Service Research Ethics Committee up to age 14 years, and the National Research Ethics Service at age 17 years. Informed parental consent was obtained in advance of data collection up to age 14 years. At age 17 years, verbal informed consent was provided by the cohort members. Participants were able to refuse to participate in any element of data collection or withdraw from the study at any time.

### Stratification and sampling weights

The Millennium Cohort Study was designed to be representative of the UK population, and to provide usable data for subgroups of children.^20^ These subgroups included children from each of the four countries in the UK (England, Scotland, Wales, and Northern Ireland), children living in advantaged and disadvantaged circumstances, and children of minority ethnic groups (only in England). The Millennium Cohort Study adopted methods of random selection in areas of the UK stratified by the above criteria.^20^ Oversampling took place in the disadvantaged and minority ethnic stratum.

### Body composition

Body fat percentage (FM %) was collected at years 7, 11, 14, and 17 through foot-to-foot bio-electrical impedance analysis (BIA), using Tanita (Bf-522W; Tokyo, Japan) scales, carried out by trained interviewers to standardised protocols. Height was measured before impedance and entered into the scales, along with age and total bodyweight, which was also measured by Tanita Scales. FM in kg was calculated from FM % and total weight ((FM%/100) × weight). FFM was then calculated by subtracting FM from total weight. Further information on how body composition was measured and calculated using BIA can be found in the appendix (p 8). FM and FFM were indexed to height. The index, similar to BMI, is usually calculated by dividing mass (kg) by height squared. However, use of height squared (ie, a Benn parameter [kg/m^B^] where the power used [^B^] is 2) does not completely remove the correlation between the index and height.^21^ Therefore, we calculated the Benn parameter, which ensures that the index is uncorrelated with height. The Benn parameter was calculated for each age and sex as the regression coefficient where log FM or log FFM is regressed on log height (appendix p 8). The ratio of FM to FFM was also calculated.

### Area-level and family-level socioeconomic position

Measures of SEP were recorded at 9 months, and measures at age 3 years were used for participants who enrolled later. The Index of Multiple Deprivation (IMD) was used as the area-level measure of SEP. The IMD measures relative deprivation according to Lower-layer Super Output Areas and ranks areas from most to least deprived. The IMD from each country was combined, and five categories were defined from the 20% most deprived to the 20% least deprived areas. Income was measured using the Organisation for Economic Co-operation and Development equivalised income quintiles, creating five groups representing the lowest to highest fifth of income.

### Covariates

Ethnicity was included as a covariate because levels of obesity have previously been shown to differ by ethnicity and individuals from minority ethnic groups are more likely to live in disadvantaged circumstances.^22^ Sex was included as a covariate to improve precision of estimates, as inequalities in adiposity have previously been shown to differ by sex.^11^ Parents’ education level and occupational social class were included as covariates in models where the IMD was the exposure of interest to test the independence of IMD from family measures. Occupational social class was measured by use of the National Statistics Socio-economic Classification, which categorises occupation into five groups: 1) semiroutine and routine; 2) lower supervisory and technical; 3) small employers and self-employed; 4) intermediate; and 5) managerial and professional, with an additional group for those not working. The highest occupation of the household was selected. Education was measured from the main caregiver, usually but not always a parental respondent, and the partner. Respondents were asked about their highest academic or vocational qualification, and this was converted to a National Vocational Qualification (NVQ) scale ranging from NVQ level 1 to NVQ level 5, with additional groups for overseas qualifications and no qualifications. The highest NVQ level of the household was taken. Where one parent had an NVQ level and one parent had an overseas qualification, the NVQ level was selected.

### Statistical analysis

For each of fat mass index (FMI), fat free mass index (FFMI), and FM:FFM ratio, multilevel growth curve models, with random intercepts and slopes, were used to estimate change in body composition measure from age 7 years to 17 years. These models account for the correlation between the repeated measures of body composition within the same individual. Change with age was modelled as linear, with age centred at 7 years. There were too few data points to model non-linear growth curves. IMD and income were added, separately, and an age by SEP indicator interaction was tested to assess whether social inequalities changed with age (model 1). Model 2 also included sex, and model 3 additionally included a sex by age interaction to test how changes in outcome varied by sex. A series of further models added ethnicity (model 4), plus family income, occupation of parent with highest National Statistics Socio-economic Classification, and education level, where the IMD was the exposure variable of interest (model 5). An additional model adjusted for FMI, where FFMI was the outcome of interest (model 6) since FM and FFM are correlated, and adaptive changes in lean tissue are seen with increases in fat,^23^ so it is possible that fat drives observations for FFM and therefore mediates associations. A final model (model 7) made adjustment for FMI and family income, highest parental occupation, and education level, where IMD was the exposure variable and FFMI was the outcome variable of interest. In models 3–7, a sex–SEP interaction was included to test whether inequalities differed according to sex (p≤0·05), and models 1 and 4–7 were re-run separately in girls and boys.

### Multiple imputation

As there was some missing information for income and the IMD, and for all covariates (shown in table 1), multiple imputation was adopted to maintain sample size and reduced bias attributable to missing data, under the assumption of missing at random. A total of 30 imputed datasets were obtained, and Rubin's rule was used to combine the estimates from regression models from each of the 30 datasets. Auxiliary variables (ie, housing tenure, partners BMI, combined labour status, ever breastfed, longstanding illness, self-rated financial difficulty, and main respondent's experience of depression, smoking status, and alcohol consumption) were added to the model to improve predictions. All auxiliary variables were predictors of one or more variables being imputed. Stratification characteristics (disadvantaged and minority ethnic stratum within country) were included as a covariate.

**Table 1:** Characteristics of the full sample and covariates at baseline

	**Participants (n=19 243)**
**Mean age, years**	
7	6·82 (0·39)
11	10·68 (0·48)
14	13·77 (0·45)
17	17·18 (0·34)
**Sex**	
Male	9894 (51·4%)
Female	9349 (48·6%)
Missing	0
**Ethnicity**	
White	15 638 (81·3%)
Mixed	585 (3·0%)
Indian	495 (2·6%)
Pakistani and Bangladeshi	1333 (6·9%)
Black and Black British	720 (3·7%)
Other ethnic group	299 (1·6%)
Missing	173 (0·9%)
**NVQ**	
No qualifications	2043 (10·6%)
Overseas qualification	412 (2·1%)
NVQ level 1	1167 (6·1%)
NVQ level 2	4679 (24·3%)
NVQ level 3	2991 (15·5%)
NVQ level 4	5828 (30·3%)
NVQ level 5	1146 (6·0%)
Missing	977 (5·1%)
**National Statistics Socio-economic Classification**	
Unemployed	1432 (7·4%)
Semi-routine and routine	4909 (25·5%)
Lower supervisory and technical	1552 (8·1%)
Small employers and self-employed	1078 (5·6%)
Intermediate	2374 (12·3%)
Managerial and professional	6890 (35·8%)
Missing	1008 (5·2%)
**Income**	
Lowest quintile	4580 (23·8%)
2nd quintile	4103 (21·3%)
3rd quintile	3450 (17·9%)
4th quintile	3172 (16·5%)
Highest quintile	2909 (15·1%)
Missing	1029 (5·4%)
**Index of Multiple Deprivation**	
Most deprived 20%	6017 (31·3%)
40%	4218 (21·9%)
60%	3086 (16·0%)
80%	2508 (13·0%)
Least deprived 20%	2722 (14·2%)
Missing	692 (3·6%)

Data are mean (SD) or n (%). NVQ=National Vocational Qualification.

### Role of the funding source

The Millennium Cohort Study is an Economic and Social Research Council funded birth cohort in the UK. The current work is secondary data analysis of the existing cohort data, and the funding sources had no role in the writing or preparation of the current study.

## Results

The analytical sample consisted of 15 131 individuals who had data available on body composition, with 46 157 observations (13 436 participants at age 7 years, 12 723 at 11 years, 10 829 at 14 years, and 9169 at 17 years). In the sample, 48·6% were female, and 81·3% were of White British ethnicity (table 1). At 9 months, the largest proportion of the sample were living in the 20% most deprived areas in the UK (31·3%) and in the lowest fifth of the income distribution (23·8%; table 1).

In general, average body composition and anthropometric measures increased with age. For FMI and FFMI measured using the Benn parameter, there is an overall decline between age 7 years and 11 years, and for FMI in girls again at between age 14 years and 17 years (appendix p 8). In boys, the overall ratio of FM:FFM declines across sweeps (appendix p 1). The Benn parameter is highest at age 11 for boys and ages 7 and 11 for girls for FM due to the high correlation between height and fat mass at these ages (appendix p 8). Hence, the large differences between FMI using the Benn parameter and FMI using height squared that are noted (appendix p 1). The Benn parameter for FFMI is closer to 2 at all ages than for FMI.

**Figure 1:**
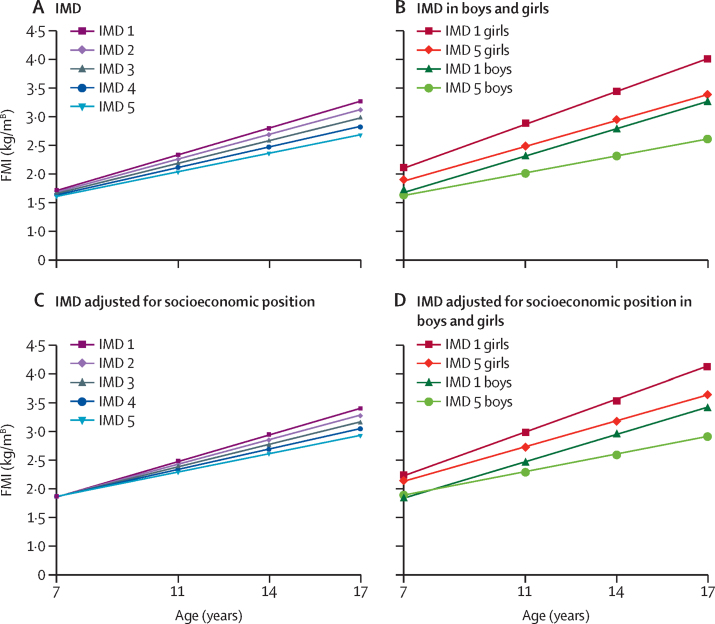
Trajectories of FMI by IMD difference in FMI (kg/m^B^) by age IMD 1 represents the 20% most deprived areas and IMD 5 represents the 20% least deprived areas. (A) Adjusted only for sex and ethnicity (model 4); (B) Model 4 in boys and girls separately; (C) Adjusted for sex, ethnicity, parental education, family income, and National Statistics Socio-economic Classification (model 5); (D) Model 5 in boys and girls separately. [B]=Benn parameter. FMI=fat mass index. IMD=Index of Multiple Deprivation.

**Figure 2:**
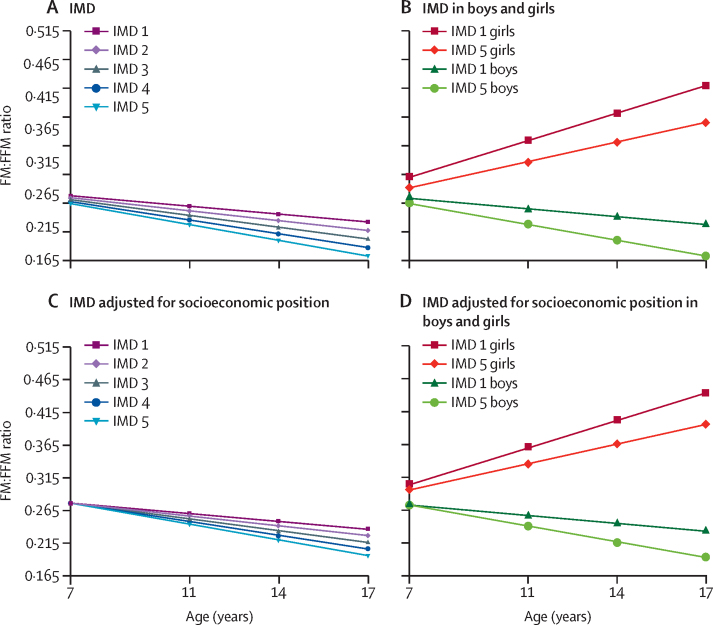
Trajectories of FM:FFM ratio by IMD Difference in FM:FFM ratio at ages 7 years, 11 years, 14 years, and 17 years by IMD group (group 1 being the most deprived group and group 5 being the least deprived group). Graph (A) adjusted only for sex and ethnicity (model 4); (B) model 4 in boys and girls separately; (C) adjusted for sex, ethnicity, parental education, family income, and National Statistics Socio-economic Classification (model 5); (D) model 5 in boys and girls separately. FM:FFM=fat mass to fat free mass ratio. IMD=Index of Multiple Deprivation.

In the unadjusted model (model 1), sex-adjusted models (models 2 and 3), and models adjusted for ethnicity (model 4), FMI and FM:FFM ratio were greater in those from more disadvantaged SEP at age 7, and inequalities increased with age (table 2), with inequalities increasing over five times for FMI and three times for FM:FFM ratio by age 17 (model 4, Figure 1, Figure 2). At 7 years, mean FMI in the most deprived IMD group was 0·10 kg/m^B^ (95% CI 0·03–0·18) higher than in the most advantaged IMD group (p=0·008), and 0·57 kg/m^B^ (0·43–0·70) higher at age 17 years (p<0·0001; model 4, figure 1A). The ratio of FM:FFM was 0·011 (95% CI 0·003–0·020) higher in the most deprived IMD group compared with the least deprived group at age 7 years (p=0·01), and 0·037 (0·026–0·047) higher at 17 years of age (p<0·0001; model 4, figure 2A). Inequalities in trajectories in FMI and FM:FFM according to income were similar to those observed for the IMD (table 2).

**Table 2:** Socioeconomic inequalities in FMI, FFMI, and FM:FFM at age 7 years, and changes in inequalities across childhood and adolescence

		**Difference in body composition at age 7 by SEP**		**SEP × age (in years) interaction**		**Age × sex interaction value**	**SEP × sex interaction p value**
		Coefficient (95% CI)	p value	Coefficient (95% CI)	p value		
**Index of Multiple Deprivation**							
FMI							
	1: Age	−0·042 (−0·058 to −0·026)	<0·0001	−0·012 (−0·015 to −0·008)	<0·0001	NA	NA
	2: Model 1 + sex	−0·038 (−0·054 to −0·022)	<0·0001	−0·012 (−0·015 to −0·008)	<0·0001	NA	NA
	3: Model 2 + age × sex	−0·028 (−0·047 to −0·010**)**	0·003	−0·012 (−0·015 to −0·008)	<0·0001	<0·0001	0·053
	4: Model 3 + ethnicity	−0·025 (−0·044 to −0·007)	0·008	−0·012 (−0·015 to −0·008)	<0·0001	<0·0001	0·05
	5: Model 4 + education + occupational social class + income	0·003 (−0·017 to 0·023)	0·76	−0·012 (−0·015 to −0·008)	<0·0001	<0·0001	0·053
FFMI							
	1: Age	−0·069 (−0·098 to −0·040)	<0·0001	0·002 (−0·003 to 0·006)	0·5	NA	NA
	2: Model 1 + sex	−0·059 (−0·88 to −0·029)	<0·0001	0·000 (−0·004 to 0·005)	0·83	NA	NA
	3: Model 2 + age × sex	−0·062 (−0·093 to −0·030)	<0·0001	0·003 (−0·001 to 0·007)	0·20	<0·0001	0·23
	4: Model 3 + ethnicity	−0·061 (−0·093 to −0·029)	0·0002	0·002 (−0·002 to 0·007)	0·25	<0·0001	0·24
	5: Model 4 + education + occupational social class + income	−0·044 (−0·077 to −0·010)	0·001	0·002 (−0·002 to 0·007)	0·25	<0·0001	0·25
	6: Model 4 + FMI	−0·041 (−0·069 to −0·013)	0·004	0·012 (0·008 to 0·016)	<0·0001	<0·0001	0·12
	7: Model 5 + FMI	−0·055 (−0·084 to −0·026)	0·0002	0·012 (0·008 to 0·016)	<0·0001	<0·0001	0·11
FM:FFM							
	1: Age	−0·004 (−0·006 to −0·002)	0·0001	−0·001 (−0·001 to −0·001)	<0·0001	NA	NA
	2: Model 1 + sex	−0·003 (−0·005 to −0·002)	0·0002	−0·001 (−0·001 to −0·001)	<0·0001	NA	NA
	3: Model 2 + age × sex	−0·003 (−0·006 to −0·001)	0·002	−0·001 (−0·001 to −0·001)	<0·0001	<0·0001	0·62
	4: Model 3 + ethnicity	−0·003 (−0·005 to −0·001)	0·011	−0·001 (−0·001 to −0·001)	<0·0001	<0·0001	0·60
	5: Model 4 + education + occupational social class + income	0·000 (−0·003 to 0·002)	0·78	−0·001 (−0·001 to 0·000)	<0·0001	<0·0001	0·61
**Income quintile**							
FMI							
	1: Age	−0·041 (−0·054 to −0·027)	<0·0001	−0·012 (−0·015 to −0·008)	<0·0001	NA	NA
	2: Model 1 + sex	−0·040 (−0·053 to −0·027)	<0·0001	−0·012 (−0·015 to −0·008)	<0·0001	NA	NA
	3: Model 2 + age × sex	−0·033 (−0·050 to −0·016)	0·0002	−0·012 (−0·015 to −0·008)	<0·0001	<0·0001	0·17
	4: Model 3 + ethnicity	−0·030 (−0·047 to −0·013)	0·001	−0·012 (−0·015 to −0·008)	<0·0001	<0·0001	0·17
FFMI							
	1: Age	−0·077 (−0·100 to −0·051)	<0·0001	0·008 (0·003 to 0·013)	0·001	NA	NA
	2: Model 1 + sex	−0·071 (−0·097 to −0·044)	<0·0001	0·006 (0·002 to 0·011)	0·006	NA	NA
	3: Model 2 + age × sex	−0·069 (−0·099 to −0·039)	<0·0001	0·007 (0·002 to 0·011)	0·003	<0·0001	0·64
	4: Model 3 + ethnicity	−0·072 (−0·100 to −0·042)	<0·0001	0·006 (0·002 to 0·011)	0·004	<0·0001	0·67
	6: Model 4 + FMI	−0·055 (−0·082 to −0·029)	<0·0001	0·017 (0·013 to 0·020)	<0·0001	<0·0001	0·67
FM:FFM							
	1: Age	−0·004 (−0·005 to −0·002)	<0·0001	−0·001 (−0·001 to −0·001)	<0·0001	NA	NA
	2: Model 1 + sex	−0·004 (−0·005 to −0·002)	<0·0001	−0·001 (−0·001 to −0·001)	<0·0001	NA	NA
	3: Model 2 + age × sex	−0·003 (−0·005 to −0·001)	0·004	−0·001 (−0·001 to −0·001)	<0·0001	<0·0001	0·29
	4: Model 3 + ethnicity	−0·002 (−0·004 to 0·000)	0·022	−0·001 (−0·001 to −0·001)	<0·0001	<0·0001	0·28

Index of Multiple Deprivation groups are ranked from 1 (lowest) to 5 (highest). Income quintiles are ranked from the lowest to the highest fifth on income. Coefficients for difference in body composition at age 7 years show the change in body composition for one unit increase in SEP. The coefficients for SEP × age interaction show the change in body composition per one unit increase in SEP per 1 year increase in age. Results presented are for models that include the SEP × sex interaction, except models 1 and 2. FMI=fat mass index. FFMI=fat free mass index. FM:FFM=fat mass to fat free mass ratio. SEP=socioeconomic position. NA=not applicable.

**Figure 3:**
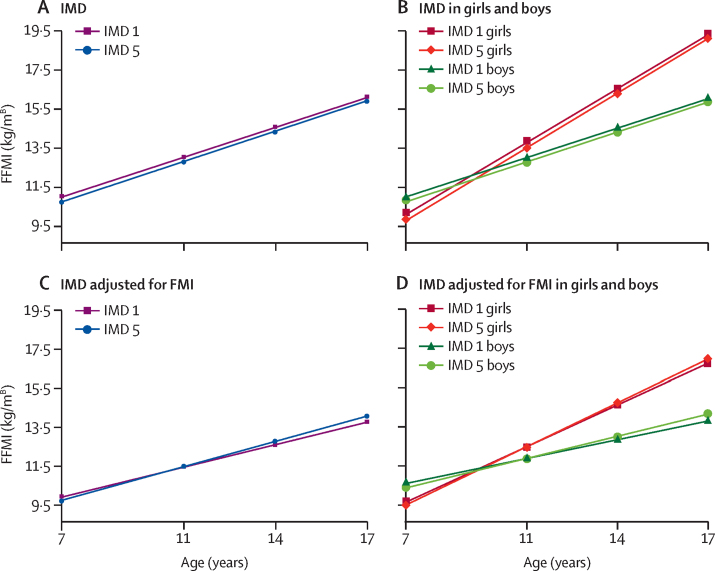
Trajectories of FFMI by IMD difference in FFMI (kg/m^B^) by age IMD 1 represents the 20% most deprived areas and IMD 5 represents the 20% least deprived areas. (A) Adjusted only for sex and ethnicity (model 4); (B) model 4 in boys and girls separately; (C) adjusted for sex, ethnicity, and FMI (model 6); (D) model 6 in boys and girls separately. [B]=Benn parameter. FMI=fate mass index. FFMI=fat free mass index. IMD=Index of Multiple Deprivation.

At age 7 years, FFMI was 0·245 kg/m^B^ higher in the most deprived IMD group (95% CI 0·118 to 0·372, p=0·0002), and the difference slightly reduced by age 17 years (model 4, appendix pp 3–4). Similar differences in FFMI were observed at age 7 years according to income but, by age 17 years, the difference between groups had decreased to 0·04 kg/m^B^ (–0·1 to 0·17, p=0·6). After adjusting for FMI (model 6), those in the most advantaged IMD groups had the fastest increase in FFMI with age. By age 17 years, the association between IMD and FFMI had reversed, so that those in the most disadvantaged IMD group had an FFMI –0·32 kg/m^B^ (–0·44 to –0·20) lower than the most advantaged IMD group (p<0·0001; figure 3C). A similar pattern was observed for income, but a larger difference between income groups was observed at 17 years (–0·44 kg/m^B^, –0·55 to –0·33, p<0·0001).

Differences were observed between boys and girls in the mean trajectories for all body composition measures as indicated by sex–age interactions. However, evidence of sex–SEP interactions was only observed for associations between IMD and FMI (table 2).

In sex-stratified models 4 and 5, inequalities by IMD in FMI were evident in girls but not in boys at age 7 years, but inequalities widened fastest in boys (table 3). In models adjusted for ethnicity (model 4) girls in the most deprived IMD group had a mean FMI 0·22 kg/m^B^ (95% CI 0·13 to 0·32) higher than those in the most advantaged IMD group at 7 years of age (p<0·0001), while the difference in boys was 0·05 kg/m^B^ (–0·04 to 0·15, p=0·3). By 17 years, the difference in FMI between groups had almost tripled in girls (0·61 kg/m^B^, 0·45 to 0·76, p<0·0001), whereas there was over an 11 times increase in the difference among boys (0·62 kg/m^B^, 0·42 to 0·82, p<0·0001, figure 1B). For income, inequalities were observed in boys at age 7 years, and widening of inequalities was similar in boys and girls (appendix p 2).

**Table 3:** Socioeconomic inequalities in FMI to FFMI and FM:FFM at age 7 according to the IMD in boys and girls to and changes in inequalities across childhood and adolescence by IMD group

		**Difference in body composition at age 7**		**IMD × age (years) interaction**	
		Coefficient (95% CI)	p value	Coefficient (95% CI)	p value
**Boys**					
FMI					
	1: Age	−0·016 (−0·040 to 0·007)	0·18	−0·014 (−0·019 to −0·009)	<0·0001
	4: Model 1 + ethnicity	−0·013 (−0·037 to 0·010)	0·26	−0·014 (−0·019 to −0·009)	<0·0001
	5: Model 4 + education + occupational social class + income	0·013 (−0·012 to 0·038)	0·31	−0·014 (−0·019 to −0·009)	<0·0001
FFMI					
	1: Age	−0·061 (−0·095 to −0·028)	0·0004	0·002 (−0·003 to 0·007)	0·47
	4: Model 1 + ethnicity	−0·063 (−0·097 to −0·029)	0·0003	0·002 (−0·004 to 0·007)	0·51
	5: Model 4 + education + occupational social class + income	−0·044 (−0·080 to −0·008)	0·017	0·002 (−0·004 to 0·007)	0·51
	6: Model 4 + FMI	−0·063 (−0·089 to −0·037)	<0·0001	0·016 (0·012 to 0·020)	<0·0001
	7: Model 5 + FMI	−0·069 (−0·970 to −0·041)	<0·0001	0·016 (0·012 to 0·020)	<0·0001
FM:FFM					
	1: Age	−0·002 (−0·005 to 0·000)	0·054	−0·001 (−0·001 to −0·001)	<0·0001
	4: Model 1 + ethnicity	−0·002 (−0·004 to 0·001)	0·15	−0·001 (−0·001 to 0·000)	<0·0001
	5: Model 4 + education + occupational social class + income	0·00 (−0·002 to 0·003)	0·76	−0·001 (−0·001 to 0·000)	<0·0001
**Girls**					
FMI					
	1: Age	−0·059 (−0·083 to −0·035)	<0·0001	−0·010 (−0·014 to −0·006)	<0·0001
	4: Model 1 + ethnicity	−0·056 (−0·080 to −0·031)	<0·0001	−0·010 (−0·013 to −0·006)	<0·0001
	5: Model 4 + education + occupational social class + income	−0·024 (−0·050 to 0·002)	0·071	−0·010 (−0·013 to −0·006)	<0·0001
FFMI					
	1: Age	−0·08 (−0·125 to −0·035)	0·001	0·004 (−0·003 to 0·010)	0·28
	4: Model 1 + ethnicity	−0·078 (−0·123 to −0·032)	0·001	0·003 (−0·003 to 0·010)	0·31
	5: Model 4 + education + occupational social class + income	−0·06 (−0·108 to −0·013)	0·013	0·003 (−0·003 to 0·010)	0·31
	6: Model 4 + FMI	−0·04 (−0·081 to 0·002)	0·061	0·010 (0·004 to 0·016)	0·001
	7: Model 5 + FMI	−0·056 (−0·099 to −0·012)	0·012	0·010 (0·004 to 0·016)	0·001
FM:FFM					
	1: Age	−0·005 (−0·007 to −0·002)	<0·001	−0·001 (−0·001 to 0·000)	<0·0001
	4: Model 1 + ethnicity	−0·004 (−0·007 to −0·002)	0·002	−0·001 (−0·001 to 0·000)	<0·0001
	5: Model 4 + education + occupational social class + income	−0·002 (−0·005 to 0·001)	0·28	−0·001 (−0·001 to 0·000)	<0·0001

IMD groups are ranked from 1 (the lowest IMD group) to 5 (the highest IMD group). Coefficients for difference in body composition at age 7 years show the change in body composition for one unit increase in IMD. The coefficients for IMD × age interaction show the change in body composition per one unit increase in IMD group per 1 year increase in age. FMI=fat mass index. FFMI=fat free mass index. FM:FFM=fat mass to fat free mass ratio. IMD=Index of Multiple Deprivation.

After adjusting models for family-level SEP (model 5), inequalities at age 7 years for FMI and FM:FFM ratio were no longer observed (table 2). However, the rate of change stayed the same as models not adjusted for family SEP, such that by age 17 years FMI is 0·45 kg/m^B^ (95% CI 0·31 to 0·59, p<0·0001) and FM:FFM ratio is 0·03 (0·016 to 0·037, p<0·0001) higher in the most deprived group compared with the most advantaged IMD group. A similar pattern is observed when looking at boys and girls separately, but with some evidence of inequalities at age 7 years in girls for FMI (table 3, figure 1D).

In model 5, inequalities in FFMI were similar but slightly smaller to those observed when only adjusted for ethnicity (model 4). When FMI was added to the model (model 7), the association between IMD and FFMI reversed by age 17 years so that there was a –0·26 kg/m^B^ (95% CI –0·39 to –0·14) lower FFMI in the most disadvantaged compared with the most advantaged IMD group (p<0·0001). Slightly larger inequalities in model 5 were observed in boys compared with girls at age 17 (model 7, appendix p 4).

## Discussion

Children and young people (aged 7–17 years) growing up in disadvantaged circumstances had higher FMI, FFMI, and FM:FFM ratio compared with their more advantaged counterparts. These differences increased with age for FMI and FM:FFM ratio. For FFMI the association reversed when adjusted for FMI. There is greater evidence of inequalities in FMI in girls in childhood, but inequalities widen at a faster rate in boys so that by late adolescence, inequalities in boys and girls are similar. Inequalities by area-level SEP remain at older ages when accounting for family-level SEP.

Consistent with our findings, previous research using the Millennium Cohort Study at ages 7 years and 11 years identified inequalities in body fat percentage that increased between the two ages.^24^ We show, using FMI, that this increase continues through to age 17 years. The Avon Longitudinal Study of Parents and Children reported a widening of inequalities in FM for girls from age 9 years to 15 years but, unlike our findings, not for boys.^25^ Our results are similar to research that has shown emerging inequalities in BMI in childhood and widening with age in the UK.^18^

Previous work shows higher FFM in those from more advantaged SEP^11^ without adjustment for FM. We show the opposite association in early childhood in all models, with advantage related to lower FFMI but, when adjusted for FMI, by age 17 years advantage is associated with higher FFMI. Previous studies have predominantly used raw or percentage measures of FFM and FM, which do not account for the contribution of height.^26^ Studies that have used indexed measures of FFM in children have found less evidence of association with SEP than those that used raw or percentage measures.^11^ Inequalities in height, which have reduced over time in the UK,^27^ are probably important in understanding inequalities in FFM.^11^ We address the correlation with height by indexing FFM using the Benn parameter.

There might be differences in inequalities by cohort, as prevalence of obesity in the Millennium Cohort Study are greater compared with previous generations, and inequalities in BMI are larger.^27^ Before adjusting FFMI for FMI, greater disadvantage was associated with higher FFMI, similar to FMI, although the difference narrowed with age as FFMI increased faster in those from more advantaged SEP. This finding could be a result of adaptive increases in muscle as a result of the higher FMI^23^ in disadvantaged children. Thus, adjusting for FMI might represent an overadjustment. Our results show that FFMI increases faster across childhood and adolescence in the more advantaged groups, resulting in increasing inequalities in the FM:FFM ratio. This result could be due to increasing inequalities in health-related behaviours that build lean mass, such as physical activity. If the more rapid increase in FMI but slower increase in FFMI observed in the more disadvantaged groups in the Millennium Cohort Study continues past adolescence resulting in continuing widening of the FM:FFM ratio, it will be increasingly difficult to address inequalities in FFM after peak FFM is reached in early to mid-adulthood.^8^ Inequalities in FFMI also add to the likely effect on inequalities in health, given the importance of FFM for later cardiometabolic disease.^5^, ^7^

While we found greater inequalities in FM among girls in childhood, boys from more disadvantaged circumstances exhibited faster rises in inequalities such that inequalities are similar between sexes by adolescence. This differs to previous research, which has shown greater inequalities in female BMI^28^ and FM^25^ in childhood and adolescence. Follow-up of childhood cohorts is needed to monitor if the observed faster widening of inequalities among males continues into adulthood. This observation might indicate a narrowing of the gap in inequalities between males and females in high-income countries in more recent generations, compared with older generations in which females typically showed greater evidence of social inequalities in obesity^1^ and body composition.^29^

Area-level measures of SEP might be related to levels of obesity due to similarity with aspects of the obesogenic environment. Our results somewhat support this by indicating that the IMD captures elements of the environment beyond family SEP in adolescence, but not in early childhood. The aspects of the environment that area-level SEP capture is unclear. It is possible that they capture the cultural and social environment, which include how social norms, networks, and peer behaviours influence diet and physical activity in adolescence.^15^ Additionally, the UK's more disadvantaged areas, as indicated by IMD, have greater fast-food density^30^ highlighting a link with the built environment. Further research is needed to understand what additional environmental factors could explain the association with area-level deprivation.

Our results show the long-term effects of disadvantage measured at age 9 months on body composition in childhood and adolescence. Family SEP in early life influences the development of nutrition and physical activity patterns and these behaviours track across childhood.^31^ There could also be clustered risk associated with disadvantage, such as social vulnerabilities,^32^ in early life; these vulnerabilites present at birth and have previously been shown to be related to obesity from age 6 years in a Spanish Cohort.^33^ Additionally, previous research shows that early life SEP is related to adult FM, but with limited and less consistent results for FFM, and no research has been conducted for the FM:FFM ratio.^29^ Continued follow-up of childhood cohorts will be needed to investigate whether these early life effects persist as with these previous cohorts, and if inequalities in adult body composition are a result of accumulation of disadvantage in childhood and adulthood. However, SEP does change over the life course, and, for area-level measures, this can be both because people move home and as areas can change around people over time and hence research into the effects of social mobility are also required.

The data are from a large, nationally representative cohort of children in the UK. We used multiple imputation to address missing data in SEP and covariates. However, there were still missing data in the outcome due to attrition of participants from the study. Body composition was only collected at four timepoints, meaning that linear growth with age was assumed. Increases in BMI are typically linear after the adiposity rebound at age 7 years and up until older adolescence.^34^ It is therefore possible that the effect estimates could be biased if the assumption of linearity does not hold.

We indexed FFM and FM for height and calculated population, age, and sex specific Benn parameters. In doing so, we removed the correlation of mass with height. However, sample sizes were not large enough to calculate the Benn parameter by ethnicity as well as sex. It was also not possible to distinguish the contribution of bone mass and lean mass using FFM measured by BIA, as compared with dual x-ray absorptiometry, BIA does not provide detailed separate measures of lean mass and bone mass. Therefore, associations could partially represent inequalities in bone density, which has previously been shown.^25^

The BIA machines were calibrated before use, used prediction equations derived from large multi-ethnic populations, and have been validated against dual x-ray absorptiometry.^35^ Although all participants were asked to remove bulky items and wear light indoor clothing, no further control measures were adopted, such as voiding or not drinking beforehand, or taking measures at the same time of day, which could have affected accuracy of measures as BIA uses water content to estimate body composition.

We make an important distinction between effects of area-level and family-level SEP and use the IMD to capture social and economic elements of the obesogenic environment, an area that has previously been understudied. However, the IMD is a broad measure of the social and economic environment that does not directly measure or distinguish aspects of the physical environment, although it has been shown to be correlated with fast-food density.^30^ These aspects of the obesogenic environment might be better captured by other measures of the physical environment, such as fast-food density and green spaces.

In conclusion, our findings indicate a less healthy body composition among children and young people growing up in disadvantaged socioeconomic circumstances and living in more deprived areas than their more advantaged counterparts. If widening of inequalities persists into adulthood, this effect will result in large differences in body composition, with subsequent consequences for health inequalities. In addition to the expected inequalities in FM, differences in childhood FFM peaks at an earlier age in the life course than does FM, meaning that inequalities established in childhood might be harder to address in later life. Our findings support the need for effective policies to tackle inequalities in obesity in childhood, but they also suggest a need to consider promotion of physical activity in disadvantaged settings from an early age to improve quality of FFM, especially muscle, and the FM:FFM ratio. Steps taken earlier will avert the need for public health interventions at later ages, where such measures might be less effective at reducing inequalities than action earlier in the life course.

## Data sharing

All data is available through the UK Data Service with an end user license.

## Declaration of interests

YK reports grants from Economic and Social Research Council (ESRC) grants from National Institutes of Health and Research, and grants from Medical Research Council, outside of the submitted work; RH reports grants from ESRC, during the study; REL reports grants from ESRC, during the study; CBS declares no competing interests.
