# Correction to *Lancet Public Health* 2021; 6: e598–607

**DOI:** 10.1016/S2468-2667(22)00114-1

**Published:** 2022-06-01

*Bridger Staatz C, Kelly Y, Lacey RE, Hardy R. Area-level and family-level socioeconomic position and body composition trajectories: longitudinal analysis of the UK Millennium Cohort Study.* Lancet Public Health *2021; **6:** e598–607*—In the Methods section of this Article, a correction has been made to the first sentence of the Area-level and family-level socioeconomic position section and the Covariates section to confirm that only sex and ethnicity variables (not socioeconomic position measures) were recorded at age 3 years for participants who enrolled after age 9 months. A new sentence has been added to the Covariates section to say: “Sex and ethnicity were recorded at 9 months, and measures at age 3 years were used for participants who enrolled later.” Additionally, in the Multiple imputation section of the Methods, we have added “mother's BMI” and “birthweight” to the list of auxiliary variables described in the third sentence. These corrections have been made as of June 1, 2022.

