# Supplementary material for: Area-level and family-level socioeconomic position and body composition trajectories: longitudinal analysis of the UK Millennium Cohort Study
Source: Lancet Public Health. 2021 Jul 29;6(8):e598–607. doi: 10.1016/S2468-2667(21)00134-1 (PMC8342403; doi:10.1016/S2468-2667(21)00134-1)
Supplement: Supplementary appendix [file mmc1.pdf]

THE LANCET
Public Health

Supplementary appendix

This appendix formed part of the original submission and has been peer reviewed. We post it as supplied by the authors.

Supplement to: Bridger Staatz C, Kelly Y, Lacey RE, Hardy R. Area-level and family-level socioeconomic position and body composition trajectories: longitudinal analysis of the UK Millennium Cohort Study. *Lancet Public Health* 2021; **6:** 598–607.

Appendix

Table 2. Mean and SD for Body Composition and Anthropometric Measure at ages 7, 10, 14 and 17

	Boys Mean (SD)				***Girls*** Mean (SD)			
	**Age 7**	**Age 11**	**Age 14**	**Age 17**	**Age 7**	**Age 11**	**Age 14**	**Age 17**
Body Composition (BIA)								
N	*6,791*	*6,428*	*5,493*	*4,587*	*6,645**	*6,295*	*5,336*	*4,582*
Fat Mass (kg)	5·37 (2·60)	8·87 (5·08)	10·68 (7·62)	12·73 (9·52)	5·90 (2·73)	11·07 (5·99)	16·27 (7·75)	18·74 (9·66)
Fat Mass %	20·17 (5·12)	20·23 (7·90)	16·89 (8·15)	16·28 (8·46)	22·26 (5·63)	24·71 (7·38)	27·15 (6·99)	28·11 (8·17)
Fat Mass Index (kg/m^2)	3·45 (1·48)	4·09 (2·48)	3·81 (2·72)	4·08 (3·04)	3·84 (1·59)	5·06 (2·52)	6·24 (2·90)	6·98 (3·53)
Fat Mass Index (Benn Index)	2·29 (0·91)	1·43 (0·82)	2·30 (1·64)	4·15 (3·09)	2·36 (0·92)	1·89 (0·91)	4·09 (1·89)	3·59 (1·82)
Fat Free Mass (kg)	20·32 (2·84)	32·01 (5·30)	47·92 (8·11)	59·03 (8·06)	19·58 (2·63)	31·17 (5·05)	40·92 (5·09)	44·34 (4·79)
Fat Free Mass %	79·83 (5·12)	79·76 (7·90)	83·11 (8·155)	83·72 (8·46)	77·73 (5·63)	75·29 (7·38)	72·85 (6·99)	71·89 (8·17)
Fat Free Mass Index (kg/m^2)	13·15 (1·12)	14·95 (1·62)	17·12 (1·90)	18·90 (2·07)	12·86 (1·00)	14·40 (1·43)	15·74 (1·41)	16·59 (1·59)
Fat Free Mass Index (Benn Index)	11·99 (1·00)	11·78 (1·22)	12·73 (1·36)	18·17 (1·99)	11·77 (0·89)	11·57 (1·11)	14·24 (1·27)	22·16 (2·06)
FM: FFM Ratio	0·26 (0·09)	0·27 (0·16)	0·22 (0·15)	0·21 (·14)	0·29 (0·11)	0·34 (0·14)	0·39 (0·14)	0·41 (0·17)
Anthropometrics**								
Weight (kg)	25·68 (4·95)	40·89 (9·86)	58·59 (13·63)	71·79 (15·48)	25·47 (4·99)	42·26 (10·38)	57·19 (12·10)	63·11 (13·71)
N	6,894	6,512	5,572	4,660	6,741	6,361	5,396	4,670
Height (m)	1·24 (0·06)	1·46 (0·07)	1·67 (0·09)	1·77 (0·07)	1·23 (0·06)	1·47 (0·07)	1·61 (0·06)	1·63 (0·06)
N	6,930	6,594	5,642	4,723	6,758	6,489	5,622	4,850
Body Mass Index (kg/m$^{2)}$	16·60 (2·32)	19·05 (3·59)	20·95 (4·04)	23·00 (4·62)	16·70 (2·41)	19·46 (3·76)	21·99 (4·19)	23·58 (4·84)
N	6,894	6,511	5,572	4,656	6,740	6,360	5,396	4,668
Body Mass Index (Benn Index)	14·00 (1·87)	12·47 (2·22)	15·34 (2·93)	22·86 (4·59)	13·88 (1·89)	12·91 (2·38)	18·43 (3·50)	25·14 (5·15)
N	6,894	6,511	5,572	4,656	6,740	6,360	5,396	4,668

Footnote: * Sample size is n=6,646 for fat mass percentage (FM%) in girls at age 7 because one observation had missing data for weight but not FM%. **Samples size for weight, height, and body mass index are reported individually as they vary between measures at each age.

Table 5. Socioeconomic inequalities in Fat Mass Index (FMI), Fat Free Mass Index (FFMI) and Fat Mass to Fat Free Mass (FM:FFM) Ratio at age 7 according to income in boys and girls, and changes in inequalities across childhood and adolescence .

	Difference in body composition at age 7 by Income Quintile (1: lowest income group, to 5: highest income group)		*Income Quintile x Age (Years) Interaction*	
Model	**Coefficient (95% CI)**	**p Value**	**Coefficient (95% CI)**	**p Value**
**BOYS**				
**Fat Mass Index**				
1: Age	-0·032 (-0·052, -0·012)	0·002	-0·012 (-0·017, -0·007)	<0·001
4: Model 1 + Ethnicity	-0·029 (-0·049, -0·009)	0·005	-0·012 (-0·017, -0·007)	<0·001
**Fat Free Mass Index**				
1: Age	-0·075 (-0·11, -0·045)	<0·001	0·008 (0·002, 0·013)	0·006
4: Model 1 + Ethnicity	-0·08 (-0·11, -0·05)	<0·001	0·007 (0·002, 0·013)	0·008
6: Model 4 + FMI	-0·074 (-0·098, -0·051)	<0·001	0·021 (0·017, 0·026)	<0·001
**Fat Mass to Fat Free Mass Ratio**				
1: Age	-0·002 (-0·005, 0·00)	0·022	-0·001 (-0·001, -0·001)	<0·001
4: Model 1 + Ethnicity	-0·002 (-0·004, 0·00)	0·11	-0·001 (-0·001, -0·001)	<0·001
**GIRLS**				
**Fat Mass Index**				
1: Age	-0·045 (-0·065, -0·026)	<0·001	-0·012 (-0·016, -0·009)	<0·001
4: Model 1 + Ethnicity	-0·042, (-0·062, -0·023)	<0·001	-0·012 (-0·016, -0·008)	<0·001
**Fat Free Mass Index**				
1: Age	-0·071 (-0·11, -0·03)	0·001	0·006 (-0·001, 0·012)	0·11
4: Model 1 + Ethnicity	-0·072 (-0·11, -0·031)	0·001	0·005 (-0·001, 0·012)	0·12
6: Model 4 + FMI	-0·044 (-0·081, -0·006)	0·022	0·015 (0·009, 0·021)	<0·001
**Fat Mass to Fat Free Mass Ratio**				
1: Age	-0·004 (-0·006, -0·002)	<0·001	-0·001 (-0·001, -0·001)	<0·001
4: Model 1 + Ethnicity	-0·004 (-0·006, -0·002)	<0·001	-0·001 (-0·001, -0·001)	<0·001

Footnotes: Coefficients for difference in body composition at age 7 show the change in body composition for one unit increase in income quintile at age 7. The coefficients for Income x Age Interaction show the change in body composition per one unit increase in income quintile per one year increase in age. Abbreviations: FMI **–** Fat Mass Index; 95% CI – 95% Confidence Intervals.

Appendix Figure Legends

Figure 3ii. Trajectories of Fat Free Mass Index (FFMI) by Index of Multiple Deprivation (IMD)

Difference in FFMI (kg/m2) at ages 7, 11, 14 and 17 by index of multiple deprivation group. Graph e) Adjusted for sex, ethnicity, FMI, parental education, family income, NS-SEC (model 7); f) Model 7 in boys and girls separately.

Figure 3ii. Trajectories of Fat Free Mass Index (FFMI) by Index of Multiple Deprivation (IMD)

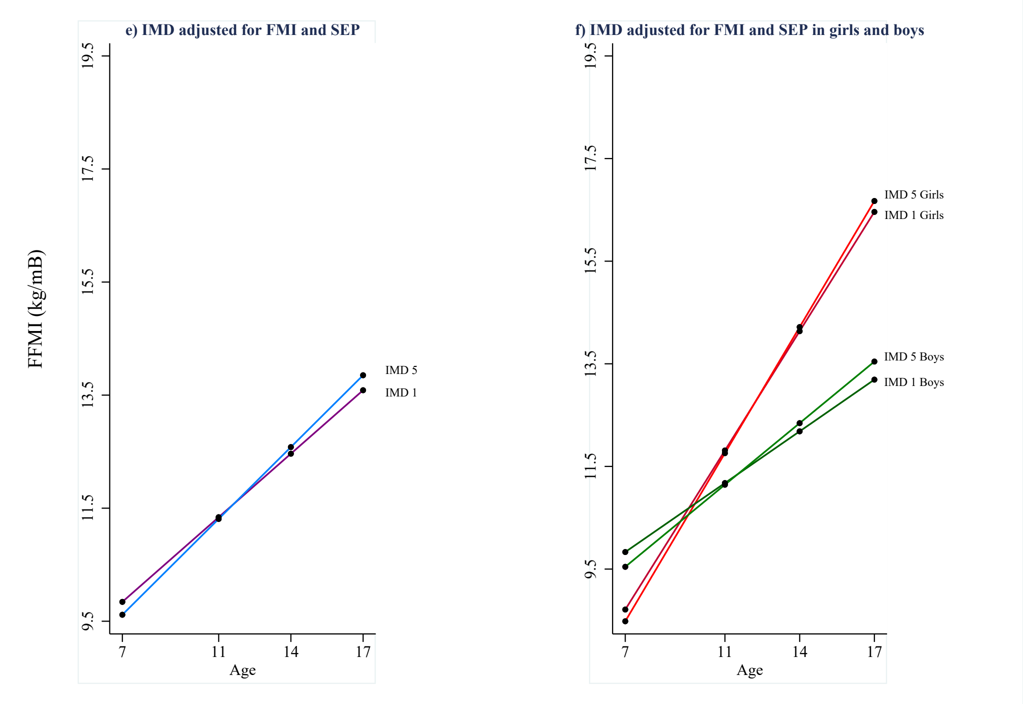

Supplementary Material 1. Measuring and Calculating Body Composition with Bioelectrical Impedance Analysis (BIA)

Body composition was measured using battery powered Tanita Scales (Bf-522W) [1-4], that comes with a handheld console and screen. Weight was measured to the nearest 0.1kg and body fat to the nearest 0.1%. Scales were calibrated before use, and placed on a firm uncarpeted surface where possible, otherwise this was noted by the interviewer. Cohort members that were pregnant or had an internal electric device were unable to have their body fat measured. For measures of body fat, cohort members were asked to wear light indoor clothing, to remove any bulky items such as watches and belts, and to remove socks and shoes. Age, height, and gender were inputted directly into the scales to allow correct calculation of fat mass percentage (FM%). Cohort members were asked to step onto scales with feet in correct position, to allow the electric current to send around their body through their feet. Equations used to calculate FM% were those used by the manufacturer [5], and were derived from large, multi-ethnic population studies. Readings from Tanita Scales have been found to correlate highly with both dual x-ray absorptiometry (used as the reference model) and Hydrodensitometry [5, 6].

Tanita Scales (Bf-522W) only provide an estimate of FM%. However, fat mass index (FMI) and fat free mass index (FFMI) are preferred to raw or percentage measures. Raw measures do not take into account the influence of body size. Percentage measures of fat mass (FM) and fat free mass (FFM) cannot be interpreted independently of each other and include the weight of FM or FFM in both the denominator and numerator when calculating the percentage, meaning that substantial changes in FM and FFM only result in small changes in the percentage measure [7]. Using FM%, along with total body weight (BW) and Height (Ht), other body composition metrics, including FMI and FFMI, are able to be calculated using the following equations [8, 9]:

FM (kg) = (FM%/100) x BW (kg)

FMI = FM (kg) / Ht (m) B

FFM = BW (kg) – FM (kg)

FFMI = FFM (kg) / Ht (m) B

FM:FFM Ratio = FM / FFM

References

1. Gray, J.C., et al., *Millennium Cohort Study: Sweep 4. Technical Report*. 2010: NatCen.
2. Gallop, K., et al., *Millennium Cohort Study Fifth Sweep (MCS5) - Technical Report*. 2013, Centre for Longitudinal Studies, Institute of Education: Ipsos MORI.
3. CLS, *Millennium Cohort Study Sixth Sweep (MCS6) - Technical Report*. 2017, Centre for Longitudinal Studies, UCL Institute of Education: Ipsos MORI.
4. CLS, *Millennium Cohort Study Seventh Sweep (MCS7) - Technical Report*. 2019, Centre for Longitudinal Studies, UCL Institute of Education Ipsos MORI.
5. TANITA. *Tanita: Frequently Asked Questions* 2021 [12/05/2021]; Available from: https://tanita.eu/help-guides/f-a-q/.
6. Barreira, T.V., A.E. Staiano, and P.T. Katzmarzyk, *Validity assessment of a portable bioimpedance scale to estimate body fat percentage in white and African-American children and adolescents.* Pediatr Obes, 2013. **8**(2): p. e29-32.
7. Wells, J.C., *Toward body composition reference data for infants, children, and adolescents.* Adv Nutr, 2014. **5**(3): p. 320S-9S.
8. Griffiths, L.J., et al., *Objectively measured physical activity and sedentary time: cross-sectional and prospective associations with adiposity in the Millennium Cohort Study.* Bmj Open, 2016. **6**(4).
9. Sera, F., et al., *Effects of physical activity on reduction of adiposity across the entire obesity distribution in primary school-aged children: findings from the Millennium Cohort Study.* Lancet, 2013. **382**: p. 89-89.

Supplementary Material 2. Estimates of the Benn Parameter for FMI and FFMI in the Millennium Cohort Study

Indexed measures are calculated using the Benn parameter (kg/mB), where the power used (B) is often two. However, a Benn parameter of two does not remove the correlation with height, as the parameter differs by age, sex, ethnicity and population. To calculate the value which ensures zero correlation between the index and height, log FM or FFM is regressed on log height, and the coefficient is used. For analysis, FMI and FFMI were both calculated using the Benn index, with the parameters calculated separately at each age and by gender as shown.

**Sex**	***Body Composition***	**Age**	**Benn Parameter**
Males	***FMI***	Age 7	3·88
		Age 11	4·76
		Age 14	2·99
		Age 17	1·97
	FFMI	Age 7	2·43
		Age 11	2·63
		Age 14	2·58
		Age 17	2·07
Females	***FMI***	Age 7	4·31
		Age 11	4·55
		Age 14	2·89
		Age 17	3·35
	FFMI	Age 7	2·43
		Age 11	2·57
		Age 14	2·21
		Age 17	1·41
